# Functional Ultra-High Molecular Weight Polyethylene Composites for Ligament Reconstructions and Their Targeted Applications in the Restoration of the Anterior Cruciate Ligament

**DOI:** 10.3390/polym14112189

**Published:** 2022-05-28

**Authors:** Sonia B. Wahed, Colin R. Dunstan, Philip A. Boughton, Andrew J. Ruys, Shaikh N. Faisal, Tania B. Wahed, Bidita Salahuddin, Xinying Cheng, Yang Zhou, Chun H. Wang, Mohammad S. Islam, Shazed Aziz

**Affiliations:** 1 School of Biomedical Engineering, University of Sydney, Sydney, NSW 2006, Australia; colin.dunstan@sydney.edu.au (C.R.D.); philip.boughton@sydney.edu.au (P.A.B.); andrew.ruys@sydney.edu.au (A.J.R.); teresa.cheng@unsw.edu.au (X.C.); 2 ARC Centre of Excellence for Electromaterials Science & Intelligent Polymer Research Institute, Australian Institute of Innovative Materials, University of Wollongong, Wollongong, NSW 2522, Australia; shaikh@uow.edu.au; 3 Department of Pharmacy, Jahangirnagar University, Savar 1342, Bangladesh; tania.wahed@juniv.edu; 4 School of Chemical Engineering, The University of Queensland, Brisbane, QLD 4072, Australia; b.salahuddin@uq.edu.au; 5 School of Mechanical and Manufacturing Engineering, University of New South Wales, Sydney, NSW 2052, Australia; ynag.zhou@unsw.edu.au (Y.Z.); chun.h.wang@unsw.edu.au (C.H.W.); m.s.islam@unsw.edu.au (M.S.I.)

**Keywords:** ultra-high molecular weight polyethylene, ligament, tendon, surface modification, biofunctionalization, synthetic graft

## Abstract

The selection of biomaterials as biomedical implants is a significant challenge. Ultra-high molecular weight polyethylene (UHMWPE) and composites of such kind have been extensively used in medical implants, notably in the bearings of the hip, knee, and other joint prostheses, owing to its biocompatibility and high wear resistance. For the Anterior Cruciate Ligament (ACL) graft, synthetic UHMWPE is an ideal candidate due to its biocompatibility and extremely high tensile strength. However, significant problems are observed in UHMWPE based implants, such as wear debris and oxidative degradation. To resolve the issue of wear and to enhance the life of UHMWPE as an implant, in recent years, this field has witnessed numerous innovative methodologies such as biofunctionalization or high temperature melting of UHMWPE to enhance its toughness and strength. The surface functionalization/modification/treatment of UHMWPE is very challenging as it requires optimizing many variables, such as surface tension and wettability, active functional groups on the surface, irradiation, and protein immobilization to successfully improve the mechanical properties of UHMWPE and reduce or eliminate the wear or osteolysis of the UHMWPE implant. Despite these difficulties, several surface roughening, functionalization, and irradiation processing technologies have been developed and applied in the recent past. The basic research and direct industrial applications of such material improvement technology are very significant, as evidenced by the significant number of published papers and patents. However, the available literature on research methodology and techniques related to material property enhancement and protection from wear of UHMWPE is disseminated, and there is a lack of a comprehensive source for the research community to access information on the subject matter. Here we provide an overview of recent developments and core challenges in the surface modification/functionalization/irradiation of UHMWPE and apply these findings to the case study of UHMWPE for ACL repair.

## 1. Introduction

The biomaterials used as biomedical implants are expected to be biocompatible such as they need to be non-toxic, non-inflammatory, and should not cause any allergic reactions in the human body [1]. Moreover, the material must have an excellent combination of high strength and low Young’s modulus closer to the implant to ensure longer service life and avoid implant loosening and revision surgery [2]. Ultra-high molecular weight polyethylene (UHMWPE) is distinguished by its high ultimate tensile strength, good biocompatibility, corrosion resistance, low water uptake, low coefficient of friction, and high abrasion resistance [3]. Such properties define UHMWPE’s use in many development areas and in medicine and biology, including the manufacture of artificial joints and implants for orthopedic surgery. All knee replacements and 85% of hip replacements today use UHMWPE on their bearing surfaces, which represents over two million orthopedic implants per year [4,5]. Two key factors decide the quantity and consistency of cell adherence to the implants: implant wettability (surface chemistry) and surface topography (surface roughness) [6,7]. Currently, UHMWPE is commercially fabricated under several brand names: Polymin SK (BASF, Ludwigshafen, Germany), Polystone M (Roechling, Mannheim, Germany), Tivar (Quadrant, Tielt, Belgium), Tecafine PE10 (Ensinger, Nufringen, Germany), Okulen 2000 (SP-Plast, Helsinki, Finland), GUR (Tina, Solidurraz, Württemberg, Germany), and by various companies, such as Goodfellow (Huntingdon, United Kingdom) and Braskem (São Paulo, Brazil, Brazilian Chemicals) [8].

In ACL and other ligament and tendon reconstructions, UHMWPE fiber is selected because it is one of the most durable materials known in the biomedical field [9,10]. In addition, it possesses excellent tensile strength, enough to support human load-bearing demands [11]. Despite these features, particular drawbacks have been noted, such as UHMWPE fibers being problematic to bond to most materials due to their chemical inertness and poor wear resistance. Wear debris generated during joint motions could cause osteolysis and implant displacement, contributing to the primary reason for joint revision [12].

UHMWPE fibers’ appealing physical and mechanical qualities are related to their highly aligned crystalline microstructure polythene chains [13]. Currently, gel-spinning processes are usually used to manufacture UHMWPE filaments. In this technique, an oxygen-rich slim limit layer is created during the turning of UHMWPE filaments, which is responsible for decreasing the bond properties of strands [14]. As a result, eliminating oxygen-rich boundaries is required to maximize fiber adhesion to other materials through surface modification of UHMWPE. Multiple methods have been utilized to modify the surface biocompatibility and wear resistance of UHMWPE [14,15]. These modifications can be divided into two types: chemical and dry techniques. Chemical surface modifications were conducted with oxidative acid etching [14], coating treatment [16,17], and chemical grafting of UHMWPE [18,19,20]. Dry surface modification techniques include different types of plasma treatments, grafting, and UV and gamma irradiation treatments [21,22,23]. Typically, molecular modification processes involve the insertion of oxygen-rich functional groups on the surface of UHMWPE fibers, which provide excellent chemical bonding sites. Additionally, the surface treatment would introduce imperfections or roughening, such as micro-pits, which act as mechanical anchor points, facilitating mechanical interlocking of the polymer matrix to fibers. Sometimes, combined methods are applied to improve interfacial adhesion of the materials [24]. Nano-reinforcement, such as carbon nanotubes (CNTs), nano clay, graphene, boron carbide, nano alumina (Al_2_O_3_), and vitamin C, has recently been employed to change the polymer matrix to be used with fiber in order to create the best potential interfacial connection through resonance [25,26,27,28].

The improvement of its surface can modify its biological and tribological properties [29,30]. The use of these materials can improve the surface hardness and abrasion resistance of the UHMWPE. Traditional ways of upgrading the wear performance of the UHMWPE include techniques such as gamma or electron beam radiation followed by thermal stabilization [31]. These techniques are accompanied by an increase in bulk mechanical properties, such as toughness, tensile strength, fatigue performance, and wear resistance [32].

Plasma treatment is currently another technologically successful and safe method (which does not need any corrosive reagents/solvents) for the surface modification of polymeric material. Properties can be improved by surface treatment of UHMWPE with argon plasma, cold atmospheric plasma (CAP), dielectric barrier discharge (DBD) plasma, plasma-assisted chemical vapor deposition (PACVD), and plasma immersion ion implantation (PIII) methods followed by protein immobilization. To resolve the issue of wear and to enhance the life of UHMWPE as an implant, in recent years, this field has witnessed numerous innovative methodologies such as biofunctionalization or high temperature melting of UHMWPE to enhance its toughness and strength. The modifications to the surface of the material through plasma can improve its hydrophilicity, surface energy, and wear resistance by introducing functional groups to the material which have been characterized by water contact angle, Fourier Transform Infrared (FTIR) and Scanning Electron Microscope (SEM) [33,34].

**Figure 1:**
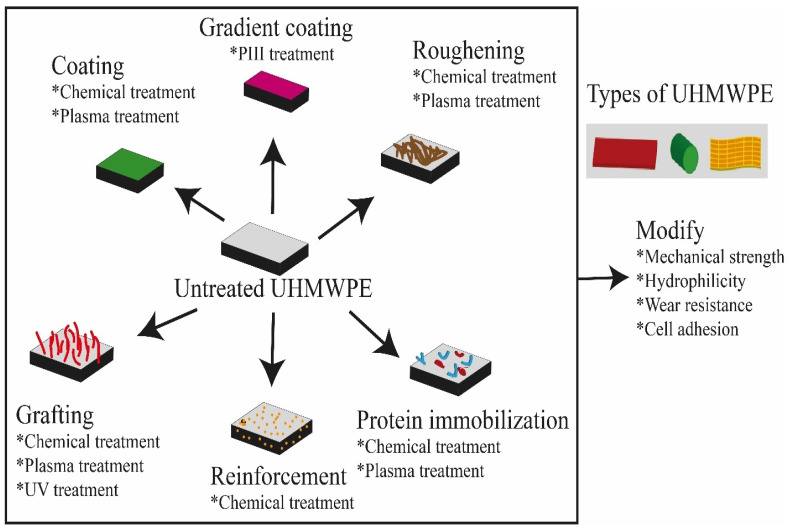
Contrasting approaches for surface functionalization of UHMWPE.

The surface functionalization/modification/treatment of UHMWPE is very challenging in orthopedic applications such as ligament regeneration. In spite of these difficulties, several surface roughening, functionalization, and irradiation processing technologies have been developed and applied in the recent past [35,36,37,38]. The basic research and direct industrial applications of such material improvement technology are very significant, as evidenced by the significant amount of open literature [39]. However, the available literature on the research methodology and techniques related to material property enhancement and protection from wear of UHMWPE is disseminated and there is a lack of a comprehensive source for the research community to access information on the subject matter. Therefore, the objective of this review is to provide an overview of recent developments and core challenges in the surface modification/functionalization/irradiation of UHMWPE and apply these findings to the case study of UHMWPE for ligament, e.g., anterior cruciate ligament, reconstruction. Figure 1 illustrates the overview of the surface treatments of UHMWPE and Table 1 summarizes the influence of surface properties on UHMWPE after surface treatments.

## 2. Background of UHMWPE as Orthopedic Implants

UHMWPE belongs to a subgroup of thermoplastic polyethylene (PE) that is obtained from monomers of ethylene via a polymerization reaction. It is composed of extremely long polyethylene chains which effectively transfer load and provide a polymer backbone by reinforcing intermolecular interactions [57]. The desired degree of polymerization of UHMWPE is dependent on its end applications, the degree of polymerization is observed in orthopedic applications within a range of 71,000–214,000 with a molecular weight ranging from 2 to 6 million g/mole [58,59]. UHMWPE is a semicrystalline polymer, and its properties are strongly dependent on its microstructure [60]. The semicrystalline structure of UHMWPE consists of two phases known as crystalline and amorphous phases. Its properties are determined by the relations between amorphous and crystalline phases, such as binding molecules, crystallinity, degree of crosslinks and entanglements, and the crystallite positions [61]. The crystalline phase comprises lamellae consisting of strongly directed folded chains [62]. UHMWPE is also known as high modulus PE or high-performance PE because of its toughness and good impact strength. High density polyethylene (HDPE) has also been used for biomedical skeletal and orthopedic applications [63]. It also has extraordinary properties such as nontoxicity, high resistance to corrosive chemicals, and wear strength that makes it reliable for orthopedic applications, but UHMWPE is more abrasion and wear resistant than HDPE. Table 2 represents the physical properties of HDPE and UHMWPE. Several monomer units attach during polymerization based on metallocene catalysts to make UHMWPE stronger compared to HDPE.

In 1962, Sir John Charnley introduced UHMWPE ($-[CH_{2}-CH_{2}{]}_{n}-$) for biomedical use, and it was then applied as a joint surface load bearing material for hip and knee replacements. Hip and knee replacements are prosthetic joints that replace human joints affected by arthritis. The oxidation resistance of UHMWPE was improved by cross-linking, high-pressure crystallization, and introducing antioxidants.

UHMWPE can also be used as woven, knitted, or nonwoven sheets to provide three-dimensional structures for cell ingrowth. UHMWPE fabrics can be produced by a gel spinning technique that allows for the parallel orientation of the fibers resulting in a high modulus of elasticity and strength. The market demand for medical-grade UHMWPE has risen tremendously from 60.9 kilotons (2015) to a projected 204.8 kilotons in (2024), according to a survey conducted by Grand view research [64]. Extensive use of UHMWPE in the medical field is due to its superior biocompatibility, chemical resistance, low wear volume, ultimate tensile strength, and low coefficient of friction.

## 3. Methodologies for Surface Modification of UHMWPE

In ACL reconstruction, UHMWPE can be used in fabric or fiber form but in other biomedical sectors it can be used in sheet, rod, or powder form. UHMWPE has been used for shoulder replacements, hip arthroplasty, ankle replacements, and other joint replacements due to its high performance with low friction coefficient, high abrasion resistance, great aging resistance, and high impact strength properties. However, inertness and extreme hydrophobicity makes it inappropriate for application in shoulder and joint replacements [65]. Different methods to improve the surface properties of UHMWPE are described below:

### 3.1. Chemical Treatment

Several types of materials such as acetic acid, sulfuric acid, and chromic acid have been used to modify the surface properties of UHMWPE [66]. Silverstein et al. [40] have explored surface treatments using chromic acid, potassium permanganate, and hydrogen peroxide to reduce the smoothness of the polymer surface. These etchants were used to remove the weak outer layer of the polymer. A rough and oxidized UHMWPE surface was formed, with increased surface tension and enhanced wetting [67]. The oxidized UHMWPE contains 6:1 combination of ether and carbonyl bonds. Increased C and O components of untreated and treated UHMWPE are presented in Table 3 [40].

Firouzi et al. [68] investigated the mechanical and biological properties of UHMWPE after nylon coating. The results confirmed that nylon coating could increase toughness, maximum breaking force, and creep time. Excellent mechanical strength and wear resistance with lower cytotoxicity can suggest UHMWPE is suitable for biomedical applications.

**Figure 2:**
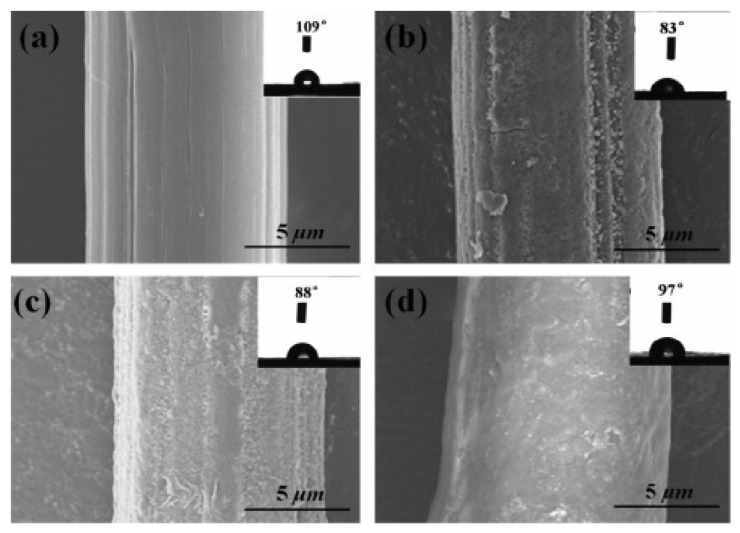
SEM images for (**a**) pristine UHMWPE fibers; (**b**) UHMWPE-PDA fibers; (**c**) UHMWPE-PDA-EDGE fibers; (**d**) UHMWPE − (PDA + EGDE) fibers; and micrographs of the water contact angle test. Reprinted with permission from [41].

**Figure 3:**
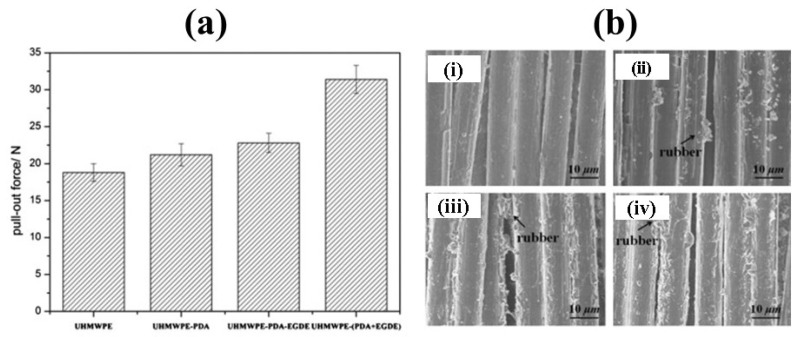
(**a**) Pull out test of UHMWPE and modified fibers; (**b**) SEM images of untreated and treated UHMWPE; (i) UHMWPE fibers/rubber, (ii) UHMWPE–PDA fibers/rubber, (iii) UHMWPE–PDA–EGDE fibers/rubber, and (iv) UHMWPE– (PDA + EGDE) fibers/rubber. Reprinted with permission from [41].

Sa et al. [69] explained another surface modification of UHMWPE fiber using bioinspired polydopamine deposition and epoxy grafting to improve the surface activity and adhesion properties of the material. One of our studies investigated wettability via the water contact angle test and tribological performance single fiber pull out test, showing the change in contact angle for untreated and modified UHMWPE (Figure 2) and increased interfacial strength of modified UHMWPE fibers compared to pure UHMWPE fiber (Figure 3) [41].

**Figure 4:**
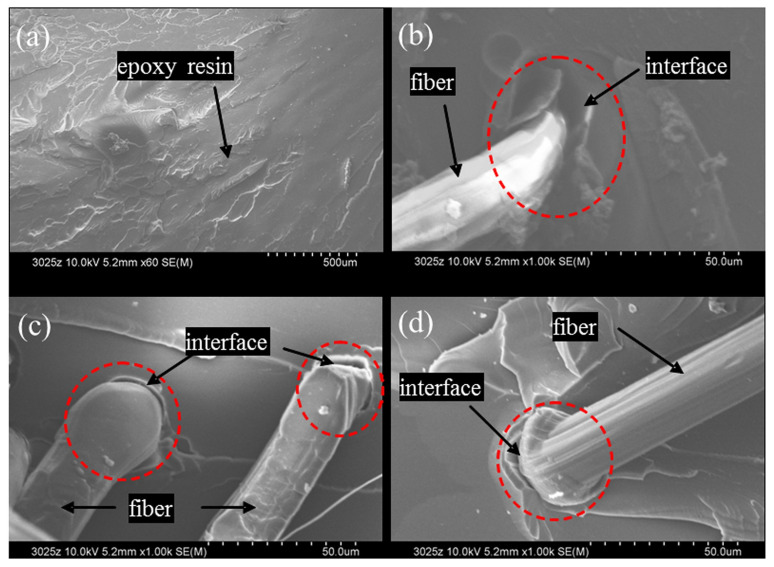
Scanning electron microscopy of UHMWPE fibers with tensile fracture surface; (**a**) epoxy resin, (**b**) UHMWPE-epoxy resin, (**c**) UHMWPE-PDA-epoxy resin, and (**d**) UHMWPE-PDA-HMDA-epoxy resin. Reprinted with permission from [42].

Similar work was carried out by J. Hu et al. [70] to improve the surface activity and adhesion property of UHMWPE fibers using polydopamine (PDA) and hexamethylenediamine (HMDA). The findings showed that the surface of UHMWPE-PDA fibers and UHMWPE-PDA-HMDA fibers were much rougher than that of pure fibers with increased interfacial shear strength. It was observed that the reaction between primary and secondary amine groups of PDA and HMDA with epoxy groups of epoxy resins increased the interfacial adhesion between the UHMWPE-PDA-HMDA with epoxy resin (Figure 4) [42].

**Figure 5:**
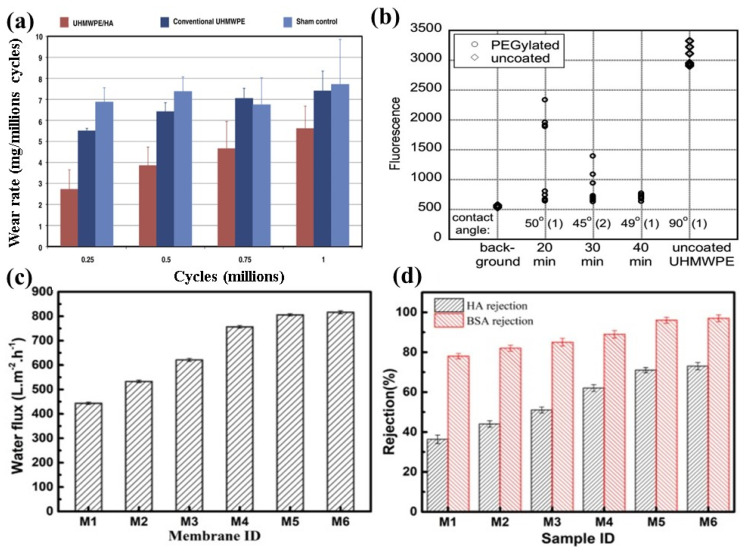
(**a**) Wear rate comparison of conventional UHMWPE and UHMWPE-HA composite and control samples; (**b**) water contact angle and protein adsorption resistance results of untreated and treated UHMWPE; water flux and protein rejection of various membranes; (**c**) Water flux; and (**d**) HA and BSA rejection. Reprinted with permission from [44,45,73].

Incorporation of HA can increase tensile strength and hydrophilicity of UHMWPE material [71,72]. James et al. [73] from Colorado State University developed a UHMWPE-hyaluronan (HA) micro composite where a small portion of HA was covalently bonded into a porous preform of UHMWPE to act as articular cartilage and joint replacement materials. It was claimed that UHMWPE is hydrophobic, while articular cartilage is hydrophilic containing chondrocytes and extracellular matrix with proteoglycans [74]. HA introduced a hydrophilic lubricous well-hydrated surface on UHMWPE. The processing of UHMWPE–HA biomaterials involves molding techniques to make it fully dense. Sham control does not include the molding step. Wear resistance of different grades of UHMWPE-HA is shown in Figure 5a. The UHMWPE-HA wear rate was slightly lower than both the UHMWPE convention and the sham control across the whole process [73].

Kane et al. [44] have examined the tribological properties of PEG-like coatings on UHMWPE for total hip replacement. PEG-like hydrogel was covalently bonded to the surface of UHMWPE to improve its lubricity, wear resistance, and antithrombogenic properties. Surface of PEGylated UHMWPE shows higher resistance to protein adsorption compared to untreated UHMWPE, and surface hydrophilicity does not have any impact on it (Figure 5b). Unlike UHMWPE particles, fragments of the PEG-like coating are less likely to induce an in vivo immune reaction.

Liu et al. [45] introduced a hydrophilic inorganic vinyl trimethoxy silane (VTMS) and SiO_2_ layer on UHMWPE to make the surface of UHMWPE more hydrophilic and to improve its antifouling properties. This hydrophilic layer will create a protective hydrated sheath by absorbing water and protecting the surface from unwanted contaminants. After the grafting, the water flux of UHMWPE increased from 452.2 L·m^−2^·h^−1^ to 532.7 L·m^−2^·h^−1^ as shown in Figure 5c. On the other hand, HA and BSA rejection of the original UHMWPE was 36% and 78%, but after the treatment it increased to 42.3% and 82% as shown in Figure 5d.

### 3.2. Surface Modification of UHMWPE by Different Plasma (DBD, PACVD, ECR, CAP, PIII) and Gamma Irradiation Methods

Plasma modification is one of the most productive techniques for surface treatment of polymers as plasma treatment could selectively modify the physical and chemical properties of the surface of the polymers without affecting the original bulk characteristics of the polymer. This treatment can modify the surface of the substrate without changing the mechanical properties of the substrate [75].

Ionized precursor fragments are deposited on the substrate surface and result in the deposition of thin films on the substrate. Plasma treatments can provide the biocompatibility of the materials [76,77], drug delivery devices [78,79], biofilms [80,81], next generations of nanobiointerfaces [82], anti-corrosion coatings, and corrosion with enhanced tribological properties, and can improve antibacterial properties [83]. Compared to other surface modification treatments, plasma surface modification offers shorter treatment times [78]. Plasma treatment is usually reliable, reproducible, non-line of sight, and applicable to different sample geometries as well as different materials such as metals, polymers, ceramics, and composites [84]. Plasma processing can provide sterile surfaces and can be adopted easily for industrial purposes. Plasma activation has been successfully performed for the improvement of the wettability of polymer surfaces [85]. Surface properties of the polymers can be modified by using energetic photons, ions, or electric beams or laser treatment. Surface modifications of UHMWPE bring changes to surface topography and wettability of the sample [86,87]. Different plasma techniques and gamma irradiation for surface modification of UHMWPE have been described below:

#### 3.2.1. Cold Atmospheric Plasma (CAP)

The functionalization of UHMWPE with cold atmospheric pressure gas plasma results in improved wear performance without affecting the cytocompatibility of the material. This is an inexpensive method that has been used for modification of the surface properties of materials and can provide a sterile surface environment. The longevity of replacement joints and discs is an important factor in determining the success of implant materials. The antimicrobial properties of cold gas plasma allow simultaneous wear performance enhancement and material sterilization [88].

**Figure 6:**
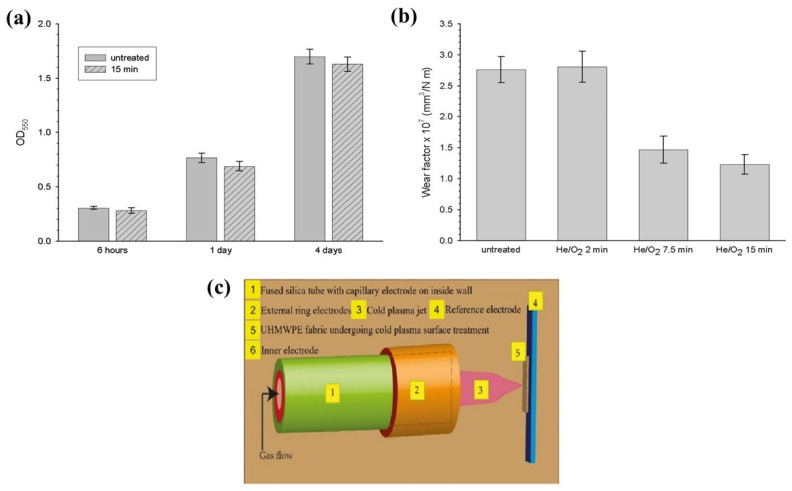
(**a**) Wear factors of untreated and treated UHMWPE after cold plasma treatment, reprinted with permission from [89]; (**b**) osteoblast cell adhesion to untreated and cold plasma treated UHMWPE, reprinted with permission from [89]; (**c**) a schematic diagram of cold atmospheric plasma.

Perni et al. [89] have determined that cold atmospheric plasma functionalized materials show a greater level of cross-linking of polyethylene chains. In this treatment, wear factors and cytotoxicity are assessed by scratch resistance and 3-(4,5-dimethylthiazol-2-yl)-2,5-diphenyl-2H-tetrazolium bromide (MTT) assay. UHMWPE is treated with He/O_2_ at different time frames. Results in Figure 6a show that wear factors of samples treated for 7 min and 15 min were almost the same but half of the untreated UHMWPE. Figure 6b shows the adhesion of osteoblast cells to UHMWPE increased over time, but there was no significant difference between untreated and treated UHMWPE.

**Figure 7:**
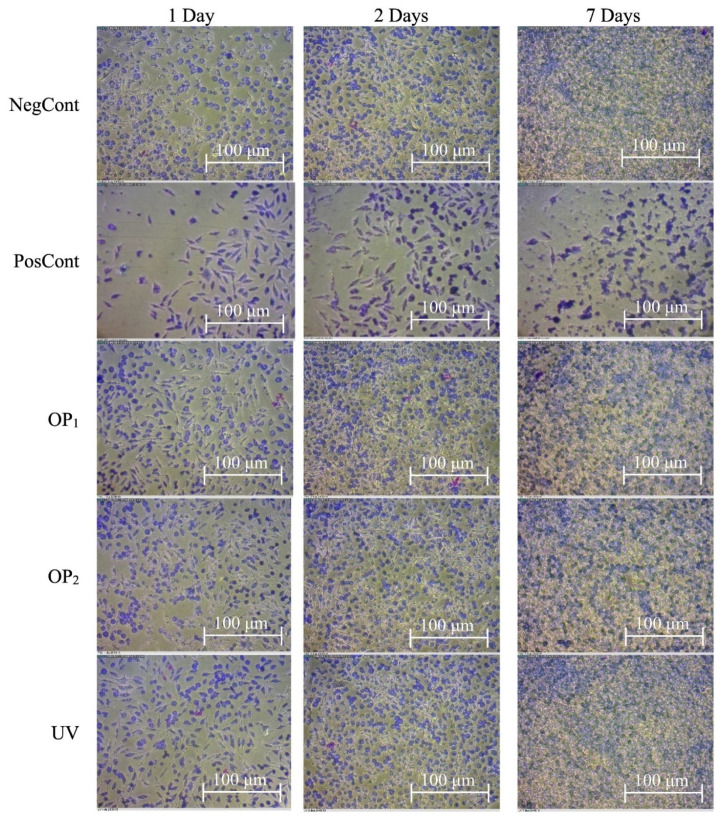
L929 cell attachment on untreated and treated UHMWPE. Reprinted with permission from [93].

In another study, S. Van et al. [90] illustrated that the surface functionalization of UHMWPE through a cold plasma method in the absence of air can provide enhanced adhesion forces, and improved hydrophilicity. Similar functionalization of UHMWPE is achieved by using cold plasma where ionized gas produced from electrical discharge penetrates the material’s surface at low pressure [91,92]. Sterilization was achieved by the formation of reactive species, which inactivates the nucleophilic sites of microorganisms. Rodrigues et al. [93] found that UHMWPE treated with cold plasma can improve hydrophilicity and influence cell adhesion properties which is illustrated in Figure 7. Cell biocompatibility was tested on three different types of treated samples (OP1-optimal points based on surface wettability, OP2, and UV). After 7 days incubation OP2 showed less cell adhesion compared to OP1 and UV. It has been confirmed from the results that hydrophobic surfaces favor cell adhesion properties more than plasma treated hydrophilic surfaces.

#### 3.2.2. Plasma-Assisted Chemical Vapor Deposition (PACVD)

**Figure 8:**
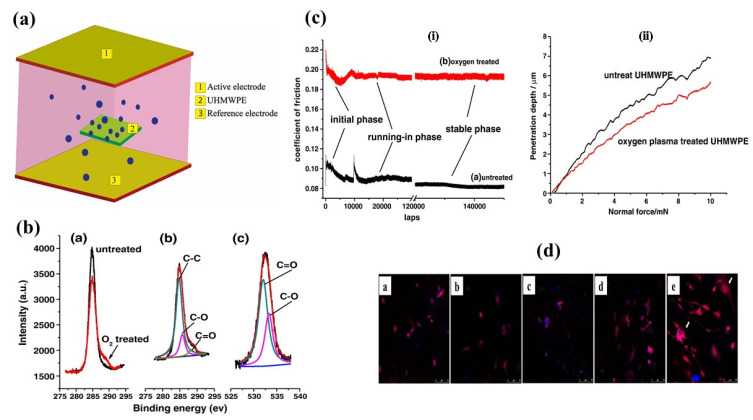
(**a**) Plasma-assisted chemical vapor deposition (PACVD) coating of UHMWPE fabric; (**b**) introduction of additional groups into UHMWPE before and after treatment, reprinted with permission from [34]; (**c**) (i) friction coefficient of friction of untreated and treated UHMWPE; (ii) scratch penetration vs. load graph, reprinted with permission from [34]; (**d**) differentiation of PBMNCs to osteoclast a. Untreated UHMWPE, b. HN-1 min, c. O_2_-1 min, and d. HN-2 min do not display presence of osteoclasts, whereas e. O_2_-2 min displays the presence of multinucleated giant osteoclasts on its surface (indicated with arrows). Reprinted with permission from [33].

Among different plasma treatments, Struszczyk et al. [22] have implemented a plasma-assisted chemical vapor deposition (PACVD) method for modification of the surface of UHMWPE composites [94]. The PACVD system consisted of two aluminum electrodes; the charged surface was positioned between the electrodes. Two materials, hexamethyldisiloxane (HMDSO) and tetradecafluorohexan, to be deposited on the UHMWPE composite were injected into the gas mixture. The surface of composite UHMWPE was modified using a low-temperature plasma method with a radio frequency of 13.56 MHz as the generator in an argon and air-gas mixture. The use of the PACVD method to deposit a polymer layer on a composite of UHMWPE can improve the main functionalities of the composite and provide new functional properties [63,95]. Das et al. [96] introduced a new technique of biomolecule immobilization on UHMWPE substrates at different gas flow rates by using the PACVD technique. This technique showed that a significant number of biomolecules attached on DLN (Diamond-like nanocomposite) coated UHMWPE rather than untreated UHMWPE. Table 4 shows adhesion strength of deposited DLN films on UHMWPE, the strength of adhesion was seen to slowly increase and then later decrease. The whole PACVD system is shown in Figure 8a.

#### 3.2.3. Electron Cyclotron Resonance (ECR) Plasma

Another technique has been developed to etch the surface of materials using gas pressure and microwave power [97]. Liu et al. [34] have studied the surface and tribological properties of UHMWPE after ECR plasma treatment. It has been stated that ECR plasma can improve wettability, anti-scratch, and tribological properties of UHMWPE through increased cross-linking of UHMWPE molecular chains. Introduction of several additional functional polar groups (C–O/C–OH and C=O group) into the material made the surface of the material hydrophilic as shown in Figure 8b. On the other hand, more cross-linking enhanced the hardness, anti-scratch, and anti-friction properties of UHMWPE which is presented in Figure 8c.

More et al. [33] presented a surface modification technique of UHMWPE with microwave-assisted electron cyclotron resonance (ECR) plasma for osteoblast and osteoclast differentiation. A microwave power of 150 W and 2.45 GHz was implanted through a quartz window into the ECR plasma chamber [98]. It has been determined that plasma treatment for 2 min increased surface roughness and reduced friction functional groups relative to untreated and 1 min plasma treated UHMWPE. This treatment plays a role in improving cell migration and facilitating the fusion of peripheral blood mononuclear cells (PBMNCs) to form osteoclasts which is shown in Figure 8d.

#### 3.2.4. Dielectric Barrier Discharge (DBD) Plasma

**Figure 9:**
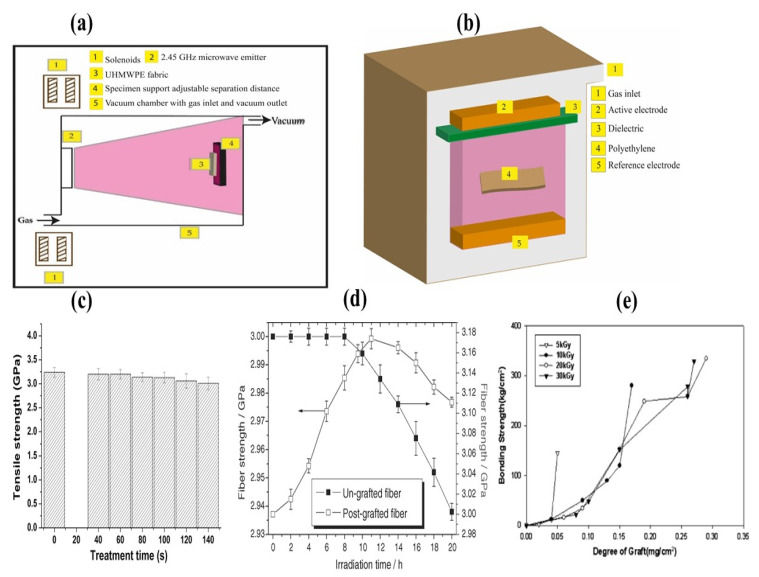
(**a**) Plasma treatment of UHMWPE fabric in an electron-cyclotron resonance (ECR) plasma reactor system. (**b**) Dielectric barrier discharge (DBD) plasma treatment of polyethylene fabric. (**c**) Impact of plasma treatment time on tensile strength of modified DBD-chitosan treatment UHMWPE fibers. Reprinted with permission from [47]. (**d**) Tensile strength of untreated and UV treated UHMWPE. Reprinted with permission from [18]. (**e**) The influence of degree of graft on tensile strength. Reprinted with permission from [99].

The Dielectric discharge barrier (DBD) is based on an alternating current (AC) discharge that produces plasma thermodynamic nonequilibrium at environmental pressure. Low temperature plasma treatments are suitable for temperature sensitive polymers. Plasma generated between two plane parallel aluminum electrodes. Electrodes were 200 cm long and the upper electrode was covered with the insulating quartz glass layer (10 mm thick). The gap width between quartz glass sheet insulation and the ground electrode was 2 mm. The sample was placed between two electrodes and exposed to plasma at 200 V, 50 Hz from mains power supply. Schematic illustrations of the DBD plasma treated apparatus is shown in Figure 9a,b.

Ren et al. [47] developed a new combined technique of dielectric barrier discharge (DBD) plasma and chitosan coatings to functionalize the surface of UHMWPE. The plasma treated sample was immersed in 0.7% *w*/*v* concentration of chitosan solution and stirred for 10 h at room temperature. Due to the plasma treatment hydroxyl, carbonyl, and carboxyl groups were introduced onto the surface of UHMWPE fiber surfaces and enhanced the wettability of the surfaces [100]. It was believed that the amino groups of chitosan could be covalently bound to the surfaces of plasma treated UHMWPE by carboxyl groups to form amide bonds and increase strength [47]. The impact of plasma treatment on UHMWPE is shown in Figure 9c.

R. Sa et al. [41] outlined two less time consuming surface modification methods to identify their effects on the surface properties of UHMWPE fibers. Three different types of epoxy resins: neat DGEBA, poly-urethane-crosslinked DGEBA, and BHHBP-DGEBA were examined as resin matrices for UHMWPE fiber-reinforced composites. Before each experiment, UHMWPE was cleaned in a polar and non-polar solvent and then dried in an oven at 60 °C. UHMWPE was plasma treated four different times, and after the treatment the fiber was exposed to the air. In the chemical method, the UHMWPE fiber was plasma treated for 10 min and then immersed in dodecyl benzyl sulfonic acid mixture at different weight ratios and temperatures. It was reported that both experiments increased the degree of interfacial contact between fibers and mechanical strength, but plasma treated UHMWPE showed better mechanical strength than chemically treated UHMWPE (Table 5) [101].

#### 3.2.5. Grafting on UHMWPE by Gamma Irradiation

Wang et al. established a surface modification technique by introducing acrylamide groups through high energy ultraviolet initiated grafting reactions, and thus, was able to increase the tensile strength of UHMWPE (Figure 9d [18]).

Diphenyl ketone was used as an initiator for absorbing energy and producing double free radicals for the process. The process of grafting was carried out in several steps: (i) initiation, (ii) propagation, and (iii) transfer and termination reactions [102]. Another similar surface modification was carried out on UHMWPE; the material was exposed to γ radiation for improving bonding strength to polymethyl methacrylate (PMMA) cement. Two types of irradiation method were adopted for this study: pre-irradiation and syn-irradiation. The intensity was 5–30 kGy for pre-irradiation and 1–3 kGy for syn-irradiation. Pre-irradiation introduced a smaller coating of PMMA on the surface of UHMWPE but higher bonding strength than syn-irradiation [99].

Hu et al. [103] studied a functionalization method for UHMWPE fabric by the radiation-induced graft polymerization reaction of γ-methacryloxypropyl trimethoxysilane (MAPS) and subsequent cohydrolysis of the graft chains (PMAPS) with tetra butyl titanate. Nanocrystalline titania films were generated on UHMWPE to improve thermal properties and UV resistance. Gamma irradiated UHMWPE exhibited a smaller area of accumulated cracks when reciprocating the loading movement compared to virgin UHMWPE. Tomita et al. [104] reported that incorporation of blended vitamin E into UHMWPE can reduce the crack formation and flaking-like destruction of pure UHMWPE [23]. Vitamin E (alpha tocopherol) blending can generate different microstructures on the surface and subsurface of the UHMWPE. Vitamin E blending reduced crystallinity which is beneficial for increasing mechanical strength [105]. It was also revealed that cross-linking and vitamin E stabilization influenced microbial adhesions on UHMWPE. The combination of vitamin E stabilization and cross-linking can give additional benefits in terms of microbial adhesion reduction [32,106].

To reduce the inertness of UHMWPE, vinyl triethoxysilane (VTEOS) containing hydrolyzable alkoxyl groups was grafted in situ onto UHMWPE by air plasma treatment. The graft proficiency is controlled by reaction conditions including time, radiofrequency (RF) plasma power, and pressure in the reaction chamber. Incorporation of VTEOS into the UHMWPE enhanced wettability, high cell proliferation rate, and coarse structure without damaging the bulk structure of the material [107]. The functionalization of biomedical UHMWPE was achieved by plasma treatment using atmospheric pressure plasma polymerization technology. The investigation conducted by Cools et al. [108] demonstrates the effect of plasma polymerization treatment on UHMWPE. They carried out the functionalization in a helium atmosphere at an ambient pressure to introduce methyl methacrylate into the PE samples to increase the adhesion between the polymer and the PMMA bone cement. The introduction of functional groups on the PE helps to anchor the polymer film to the substrate [49]. A similar study for the functionalization of UHMWPE by Aziz et al. [109] explains the post-irradiation grafting and UV polymerization onto UHMWPE surfaces. In this method of polymerization, He (Helium) has been used for activation and as a carrier gas for post-irradiation and plasma polymerization processes, and it increased the bonding strength of the material presented in Figure 9e. The treatment is based on nitrogen-containing polymer coating on UHMWPE samples by different plasma and UV based techniques at different conditions [109].

#### 3.2.6. Plasma Immersion Ion Implantation (PIII)

Plasma immersion ion implantation, commonly expressed as PIII or PI^3^, is a unique plasma treatment technique initially developed as a revolutionary non-line of sight process. Instead of other conventional ion extraction methods, PIII works by placing a 3D shaped target in the ion acceleration scheme itself [110]. In this method, the target substrate is immersed in plasma accelerated with high voltages, resulting in energetic ions being implanted from plasma to the substrate. Conrad et al. have developed the basic principles and applied them to treat specific 3D components and modified several materials using PIII [111]. Nowadays, PIII can be used to deposit a carbon-based coating on the substrate rather than as an ion bombardment surface treatment. This treatment is carried out by injecting carbon-containing gas into the plasma formed in a background gas, which is referred to as plasma immersion ion implantation and deposition (PIII and D) [112]. PIII treated diamond-like carbon films have been widely used in blood-contacting devices such as rotary blood pumps, artificial hearts, mechanical heart valves, and coronary stents [113].

In the biomedical field, one of the applications of PIII is to enhance the blood compatibility of materials [84]. Different structures and compositions of Ti-O films were synthesized by PIII treatment. The structural difference was carried out by changing the oxygen flow rate into the vacuum chamber of the PIII device. This oxygen content can change the structure of the material from amorphous to a mixed crystalline structure of anatase and rutile, and further becomes more rutile (stable form of TiO_2_). Increasing oxygen content enhanced the adhesion of platelets to the material [114,115].

Surface modification of UHMWPE by plasma immersion ion implantation with nitrogen gas showed promising results for improving surface mechanical properties such as hardness and elastic modulus [116]. Due to the formation of cross-linked molecular microstructures, the surface of UHMWPE is expected to be predominantly modified. Weak secondary bonds of the untreated material were replaced by strong covalent bonds at the cross-linked points, therefore decreasing mobility of the molecular chain. As a result, when subjected to applied force, modified UHMWPE showed higher hardness and wear resistance than the untreated UHMWPE, as shown in Table 6 [117].

Chen et al. [118] studied the surface modification of UHMWPE implanted by 80 keV N_2_^+^, C_3_H_8_^+^, with a plasma density ranging from 1 × 10^14^ to 5 × 10^15^ ions/cm^2^. Surface modification was carried out by elastic recoil detection (ERD) and X-ray photoelectron spectroscopy (XPS). Table 7 illustrated the ERD results, which detected the hydrogen deficient layer on the surface after the implantation that increased polar groups and hydrophilicity of the material.

Recently, a similar experiment was conducted by Rossi et al. [54] with a different mechanism of using surface breakdown voltage to improve the resistance of surface flashover. After the surface treatment, its surface roughness increased from 39 nm to 71 nm which has been confirmed via the AFM surface measurement technique [55]. Table 8 shows the effects of traditional parameters on surface functionalization methods. Advantages and disadvantages of different plasma methods are enlisted in Table 9.

#### 3.2.7. Nano Reinforcement

The concept of synergy, or synergistic effect, has recently evolved in the effort to attain superior adhesion and attenuate matrix dominating qualities. The fiber surface is treated with a suitable functional group, either by adding virgin nano-reinforcements or functionalized nano-reinforcements, to achieve synergistic effects. Due to the exceptional properties of nano-reinforcements such as carbon nano tubes (CNT), nano clay, multi wall carbon nanotubes (MECNT), and single wall carbon nanotubes (SWCNT), carbon nano fibers have been frequently employed to modify the polymer matrix [5,24,124,125].

Graphene nanoplatelets and CNTs are incorporated with UHMWPE to enhance its mechanical property [126,127]. Graphene is a flexible 2D carbon nanomaterial with a large specific surface area, with an ultimate tensile strength of 130 GPa. Blending of UHMWPE with graphene fillers can increase its wear resistance [128]. MWCNTs were integrated into an epoxy matrix reinforced by surface treated UHMWPE fiber to provide novel interfacial adhesion through resonance [129]. Both the UHMWPE fiber and MWCNTs were chemically treated with glycidyl methacrylate (GMA) and amino-thiol end radicals via free radical polymerization and click chemistry, respectively, to produce a high level of compatibility with epoxy matrix. LDPE/MWCNT and LDPE/MWCNT/UHMWPE self-reinforced fiber-composite were invented by M.A.A Seraji et al. [130] to improve the physical properties of UHMWPE. A novel nanocomposite coating of UHMWPE reinforced with nano clay and C nanotubes was developed to improve tribological properties of UHMWPE. This nanocomposite powder was deposited and fused onto the substrate which increased the melting temperature of the material [131].

Xin et al. [132] reported that zeolitic imidazolate frameworks (ZIF8) were mixed with carbon nano fiber to act as a filler for UHMWPE by a facile liquid-phase chemical reaction. The results show that ZIF8-CNF can greatly increase UHMWPE crystallization and improve its thermal and mechanical properties. Surface porous UHMWPE composites were invented via the hot-pressing technique, where NaCl and graphene oxide (GO) were used to enhance their mechanical and tribological properties [133].

Most recently, UHMWPE was incorporated with 0.1 wt% boron carbide by twin screw extrusion method at 200 °C to improve its physical and mechanical performance [25]. The improved wear resistance of UHMWPE was discovered to be due to increased entanglement in amorphous and increased content of the interphase (better shear resistance), as well as the load-bearing effect of the particles [134].

Biocompatible UHMWPE/nano-Al_2_O_3_/Vitamin-C hybrid composites were developed via hot press technique for joint prosthesis. Vitamin C was employed as an antioxidant and Al_2_O_3_ was used as an anti-wear additive in UHMWPE [28]. Another biomimetic composite surface produced via Polydopamine (PDA) functionalized surface and then coated with zinc oxide nanoparticles to improve interfacial adhesion properties of UHMWPE [134].

## 4. Biofunctionalization of UHMWPE by Protein Immobilization

Biomimetic materials for implants and microarray technology have been obstructed by the shortage of safe and simple methods to covalently combine bioactive molecules to the surface of a wide range of materials. Different types of wet chemical approaches are described in the literature for linking biomolecules to surfaces [135]. Puertas et al. have reported that the carboxyl group of polymers can be activated by carbodiimide chemistry [136]. N-hydroxysuccinimide (NHS), or sulfo-NHS, is also used to increase the coupling efficiency of materials [137]. Salinization and plasma functionalization have been used for pretreatment of surfaces for the generation of reactive sites for covalent coupling on polymer surfaces [138]. Biofunctionalization of the pure base material is carried out by the attachment of bioactive proteins to surfaces. Secure attachment is required, sometimes because many applications may require the protein to remain stable under hostile environmental conditions such as in the flow of blood or during washing with strong detergents or buffers [139]. Additionally, optimal device performance is dependent on dense surface coverage and long-term retention of protein conformation. Surface modification of polymer for protein attachment can be performed in physical or chemical ways, such as by attachment of linker molecules through active groups [140,141], UV treatment, plasma treatment [142], and ion beam implantation [143,144].

### 4.1. Protein Immobilization by Chemical Methods

**Figure 10:**
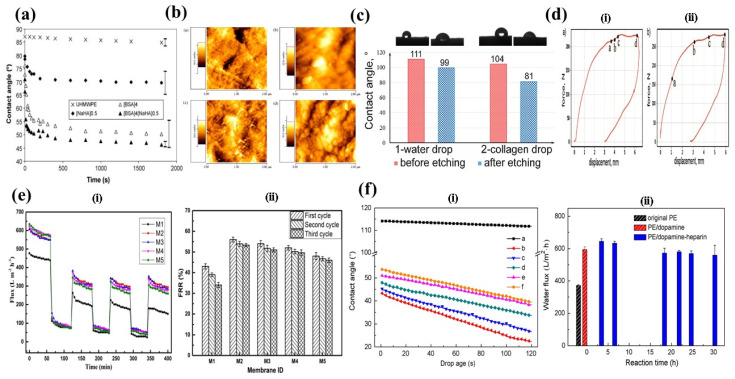
(**a**) Water contact angle of UHMWPE before and after protein incubation. Reprinted with permission from [19]. (**b**) AFM images of UHMWPE with or without protein incubation; (**a**) pure UHMWPE; (**b**) after incubation in BSA solution; (**c**) after incubation in NaHA solution; (**d**) after incubation in both solutions. Reprinted with permission from [19]. (**c**) Water contact angle of treated and untreated UHMWPE after surface treatment. Reprinted with permission from [145]. (**d**) The force displacement curve of three-point bending test for (i) freeze-dried collagen hybrid; (ii) collagen-HAp hybrid. Reprinted with permission from [145]. (**e**) Antifouling properties of different membranes (M1, M2, M3, M4, and M5): (i) variation of time-dependent flux over three periods with bovine serum albumin (BSA) as a pollutant; (ii) values of FRR with BSA as a pollutant. Reprinted with permission from [70]. (**f**) Contact angle vs. drop age curves for the original and modified PE porous membranes: (i) untreated PE membrane; (ii) PE-polydopamine composite membrane; (**c**–**f**) PE/dopamine-heparin composite membrane; (**b**) impact of heparin immobilization period for PE membranes on water flux. Reprinted with permission from [65].

Surface modification schemes are essential to functionalize the surface interfaces of materials. Serro et al. [19] examined the biotribological properties of UHMWPE after incubation in bovine serum albumin (BSA) and sodium hyaluronate (NaHA) solution. Results showed that albumin was strongly adsorbed into UHMWPE, whereas NaHA adsorbed poorly. Figure 10a shows that surfaces become more hydrophilic after protein incubation. Atomic force microscopy (AFM) results confirmed the surface morphology of UHMWPE after three different treatments. The BSA-incubated surface appeared as a globular structure, and the NaHA incubated surface exhibited a needle-like structure. Combined protein incubated surfaces had a smaller sized globular structure as shown in Figure 10b [19].

Kan et al. [145] studied the influence of collagen and hydroxyapatite (HAp) in surface functionalization of UHMWPE. It was claimed that the water contact angle dropped from 111° to 81° after collagen and HA impregnation as presented in Figure 10c. The three-point bending test plotted in Figure 10d confirmed that the increased rigidity of UHMWPE-Collagen-HAp hybrid comes from the combined impregnated layer.

PDA has been cross-linked or coated on materials to modify the surface chemistry of materials [41]. Surface modification and protein immobilization on UHMWPE polymer were carried out by dopamine self-polymerization and Schiff base reaction [70]. The dopamine layer introduced hydrophilicity of the composite membrane, and mPEG-NH_2_ modified the protein fouling resistance of the material. Figure 10e(i) shows that the pure water flux of all PDA-modified composite membranes (M2, M3, M4, and M5) remained at approximately 310 (L m^−2^ h^−1^) after three cyclic filtration tests, which was greater than that of the initial composite membrane. On the other hand, compared to the original membrane, the flux recovery ratio (FRR) values of the PDA-modified composite membranes (M2, M3, M4, and M5) were increased during all three cycles, but the FRR values of all five of these membranes were not reasonable (Figure 10e(ii)).

**Figure 11:**
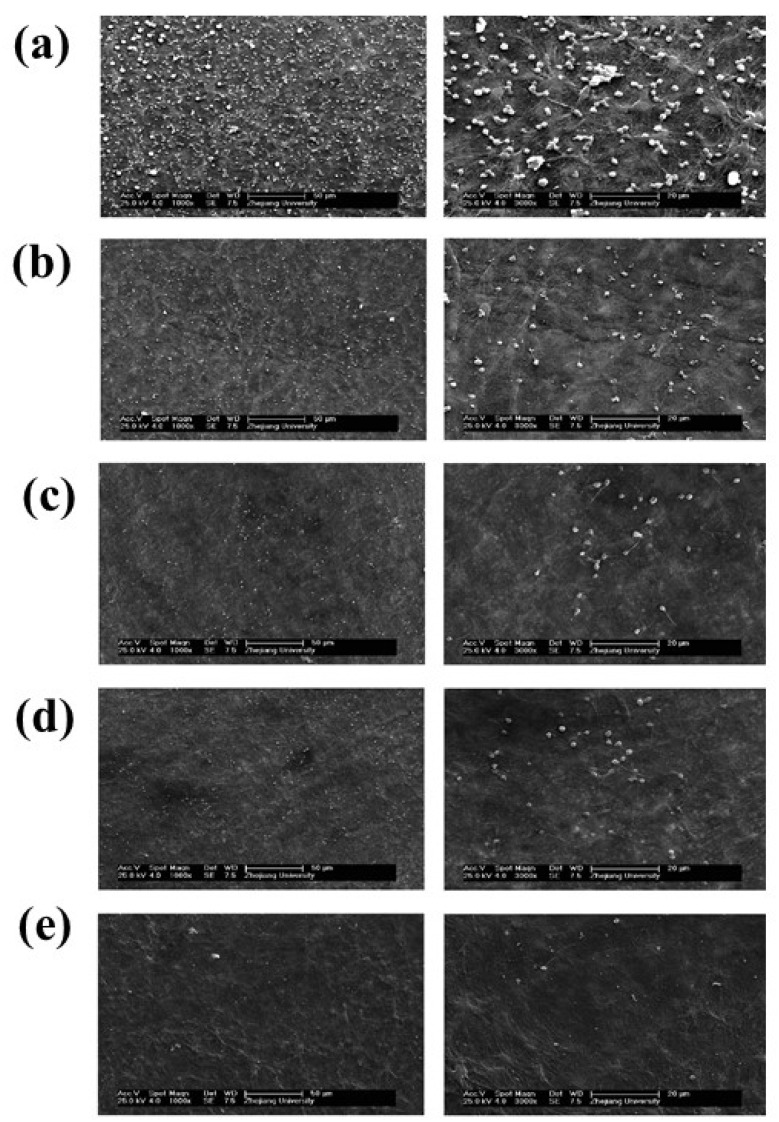
Unmodified and modified PE porous membrane surface platelet morphology: (**a**) initial PE membrane, (**b**) PE/dopamine composite membrane, (**c**,**d**) PE/dopamine-heparin composite membrane, (**c**–**e**). Reprinted with permission from [65].

In similar work conducted by Jiang et al. [65], UHMWPE was biofunctionalized for blood contacting biomedical applications with a polydopamine solution based on self-polymerization reactions. After polydopamine coating and heparin immobilization, the hydrophilicity of PE membranes was greatly enhanced. Contact angle of PE decreased drastically after surface modification with polydopamine which increased hydrophilicity of the PE membrane as shown in Figure 10f(i). Changes in water flux with retention time is presented in Figure 10f(ii). After the dopamine coating, the water flux increases 1.6 times compared to that of the original PE. Surface heparinization significantly suppressed the adhesion of platelets and enhanced the anticoagulation ability of PE membranes as observed in Figure 11. Numerous platelets acted as adhesion, outspread, and aggregated on the surface of the original PE porous membrane. On the other hand, dopamine deposition and heparin immobilization significantly reduced platelet adhesion, activation, and transformation.

### 4.2. Protein Immobilization by Plasma Methods

Radiation damage of polymers through UV treatment, plasma treatment, and ion beam implantation can provide protein binding on polymers by strong binding, especially covalent binding without specific linker groups.

Biofunctionalization of UHMWPE polymer was conducted by plasma treatment for adhesion improvement. A dielectric barrier discharge in Ar, He/O_2_, He, N_2,_ or O_2_ at atmospheric pressure was used for continuous plasma treatment of UHMWPE fibers [146]. In terms of studies related to the biomedical application of plasma-treated UHMWPE, Rodrigues et al. demonstrated that the surface treatment of UHMWPE by cold plasma techniques has significant effects on the proliferation of cells and improved the inertness of UHMWPE surfaces to a level comparable to commercially available cell culture materials [93]. Widmer and Spencer treated UHMWPE with oxygen plasma to increase its hydrophilicity to gain faster and better protein adsorption [142].

A wide range of work has focused on improving the longevity of joint implants used in human bodies, as the biomedical implant contributes to wear debris when exposed to the actions of human blood serum triggering the release of wear particles. In some studies, a dense boundary layer of human serum albumin (HSA) proteins was applied on PE surfaces to enhance boundary lubrication and reduce 50% of dynamic friction as well as reduction of static friction in hip implants [142].

The biofunctionalization of UHMWPE was derived from the detailed studies conducted by. Ho et al. explaining two methods for plasma treatment of an UHMWPE substrate. In this method of functionalization, Ar and nitrogen PIII treatment with high electrical pulses were used to modify the surface of UHMWPE to provide improved hydrophilicity and interactions with proteins. The best performance was achieved by plasma immersion ion implantation using nitrogen gas [147]. Gan et al. [148,149] reported that energetic ions extracted from inductively coupled nitrogen plasma was used to modify the surface properties of UHMWPE.

Biofunctionalization of UHMWPE was reported by Kondyurin et al., explaining the mechanism of covalently attached horseradish peroxide (HRP) on the surface of UHMWPE. The formation of the carbonized surface layer due to the oxidation of the ion damaged surface was claimed to account for protein binding on UHMWPE [147]. Influence of HRP immobilization on surface property of UHMWPE is represented in Table 10. Among various methods, ion beam implantation and its variant plasma immersion ion implantation has been used successfully for the covalent attachment of proteins on polymer surfaces [139].

Another similar approach has been made to functionalize the surface of UHMWPE by PIII treatment to improve the wettability of the surface. HRP was immobilized on plasma-treated plasticized and unplasticized PVC and UHMWPE. PIII was conducted in nitrogen plasma with a radio frequency of 13.56 MHz at different time frames. Different types of low molecular weight additives (e.g., plasticizer, solvent, adsorbed molecules on the surface) have been utilized on the surface to facilitate protein immobilization. Small molecular additives held the protein molecules and helped to attach the protein on to the surface of the polymer. However, the presence of plasticizer does not have an influence on protein attachment on UHMWPE, as presented in Table 11 [56].

**Figure 12:**
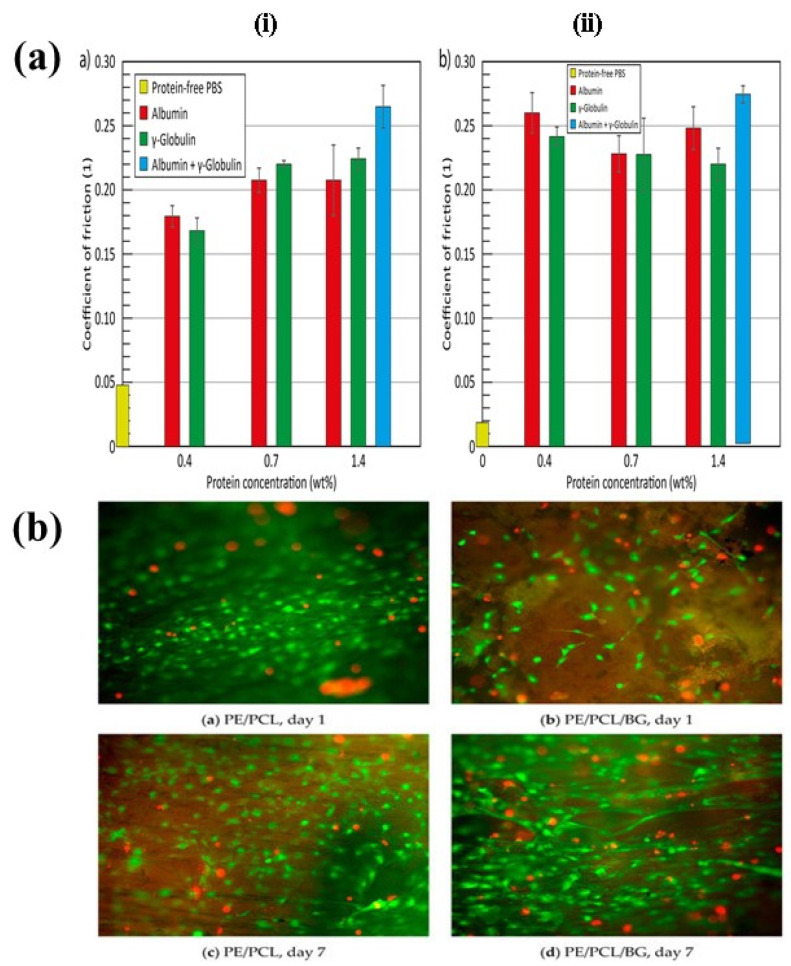
(**a**) The bar graph represents the coefficient of friction vs. protein concentration for all tested protein solutions at different sliding speed: (i) 10 mm and (ii) 50 mm. Reprinted with permission from [153]. (**b**) Live (green) and dead (red) cells on PE/PCL a,c, and PE/PCL/BG composites b,d after one a,b and seven c,d days of culture [154].

Several studies have demonstrated that biological molecules contained in synovial fluid play a significant role regarding in vivo friction and wear behavior [150]. Zolotarevova et al. [151] studied the interaction between plasma proteins and UHMWPE wear particles generated from hip periprosthetic granuloma tissues. It has been examined that UHMWPE itself is not responsible for macrophage activity. Several plasma proteins became denatured after binding with the particles and caused accumulation of macrophages and other sensitive cells. In another study, undertaken by Necas et al. [152,153], it was reported that slow sliding speed is responsible for denaturization of proteins and loss of their original structure; whereas at high sliding speed most of the protein kept their original structure and some formed aggregates. Friction behavior depends on the type of protein and its concentration. Figure 12a illustrates the correlation among the coefficient of friction, concentration of protein, and sliding speed.

## 5. Relationship between Human Ligament and UHMWPE

Ligaments are connective tissues with strong mechanical properties that can stretch a joint and become hooked at either end [155]. They attach two bones together, prevent dislocation, and restrain movement of the joints. They differ in location, size, shape, and orientation. There are four different types of ligaments in the knee, namely: medial collateral ligament (MCL), lateral collateral ligament (LCL), posterior cruciate ligament (PCL) and anterior cruciate ligament (ACL) [156,157].

### 5.1. Structure of Anterior Cruciate Ligament (ACL)

The knee joint is complex and is composed of three separate joints: the tibiofemoral, patellofemoral, and the proximal tibiofibular joints [158]. The knee joint most referred to is the tibiofemoral joint. These knee joints are stabilized by several ligaments, including the anterior cruciate ligament (ACL), the posterior cruciate ligament (PCL), the medial collateral ligament (MCL), and the lateral collateral ligament (LCL). Ligaments are made of bands of collagenous connective tissue [159]. These paralleled collagen bundles are linked to each other by cross-linking [160]. Ligaments contain two-thirds water and one-third solid. Collagen is the major component of the ligament with five prominent collagen types which are I, III, VI, XI, and XIV [161,162]. The majority (90%) of the collagen in ligaments is type I, which is responsible for its tensile strength. To maintain the mechanical and biological stability of ligaments, related organ systems, as well as the bone, play a vital role [163].

### 5.2. ACL Injury and Selection of UHMWPE for ACL Reconstruction

ACL injuries are common in sports such as football, soccer, or with uneven surfaces. ACL injuries more commonly cause knee instability that further causes injury to other knee ligaments. Injuries of the ACL range from mild, such as small tears, to severe, such as when the ligament is completely torn [164,165]. Allograft, autograft, and synthetic grafts have been used for ACL reconstruction [166]. Due to the drawbacks of allografts and autografts, synthetic grafts may be a good choice for ACL reconstruction. The synthetic ligament graft is an artificial ligament device for joining the ends of two bones. Laflamme reported that the type of material and its particle size are important factors regarding synthetic ligament graft selection [167]. UHMWPE fibers are commonly used in synthetic ligament implants due to their excellent tensile strength and elastic modulus. Mechanical properties of several materials used for ACL regeneration have been presented in Table 12.

Overen et al. [169] reported on thrombogenicity testing of UHMWPE compared to expanded polytetrafluoroethylene (ePTFE) and polyethylene terephthalate (PET) fibers for vascular applications. Hemobile is a method used to detect the damage of blood components and activation of platelets throughout the material/device. It is also used for testing vascularity of UHMWPE, ePTFE and PET fiber. Due to lower hemolysis and low activation of inflammatory responses, UHMWPE showed better hemocompatibility than ePTFE and PET fibers [169].

Hunter et al. [170] reported on attachment and proliferation of a variety of cell types on UHMWPE for orthopedic implantation. Cell multiplication was measured with a tritiated thymidine assay. Radioactively labelled thymidine (tritium) was used to measure lymphocyte proliferation by incorporation of 3H thymidine into the dividing cell’s DNA. The component vinculin, a cell focal adhesion plaque, was labeled by indirect immunofluorescence to assess the attachment of cells [171]. Fibroblasts and osteoblasts cultured directly on UHMWPE were tested by determining the mean number of adhesion plaques using an image analysis system. Fibroblasts attached well on UHMWPE fabric. High tensile strength, bio-inertness, and fibroblast adhesion makes it appropriate for ACL reconstruction [172]. Several materials have been developed that can be used for the reinforcement of the matrix to modify the properties of UHMWPE, utilized for its application in ACL reconstruction.

Surface modification plays a major role in helping osteogenesis and bone anchorage of synthetic grafts [173]. Chitosan is a naturally derived polysaccharide that has been used for the modification of synthetic grafts. Chitosan-hyaluronic is a composite that promotes new bone formation at the graft bone interface because of its porosity, biodegradability, biocompatibility, anti-infective activity, and ability to accelerate wound healing [174]. Vaquette et al. [175] reported that polystyrene sodium sulfonate as a surface modifier could improve the osteointegration of a synthetic graft [176].

Bioactive glass has been used for ligament graft modification due to its stimulation of angiogenic growth factors [177] A composite of UHMWPE-PCL-bioglass was developed as a synthetic graft using an electrospinning method. Bioglass was coated on UHMWPE via slurry dipping technique; melt derived glass particles were suspended in demineralized water to make a slurry with 5% *w*/*v* concentration, followed by 30 min agitation in a magnetic stirrer. Fibroblast cells were seeded on a composite graft to examine cell adhesion. Cells adhering to UHMWPE composite were well flattened and more spread out compared to cells on pure UHMWPE. Excellent fibroblastic cell growth on a composite UHMWPE-PCL-bioglass synthetic graft is shown in Figure 12b [154].

Bioactive glass has unique compositional ranges of dense amorphous calcium sodium phosphosilicate (CSPS) that develop strong chemical bonds with the collagen of living tissues [178,179]. The composition of 45S5 bioglass is 45% SiO_2_, 24.5% CaO, 24.5% Na_2_O, and 6% P_2_O_5_ [180]. Bioactive glass dissolves slowly in a simulated body fluid (SBF) with some reactions taking place on the surface of the glass [174,181,182,183,184]. These reactions include: (i) ions releasing due to the ion exchange between the solution and surface of the glass, but other components of the glass remain intact [185,186]; (ii) H^+^ ions attacking the silica network and as a result Si-O-Si bonds breaking down, and new Si-OH and Si (OH)_4_ groups forming at the surface of the glass; (iii) a soluble porous silica-rich layer forming on the surface of the glass due to condensation and re-polymerization; (iv) a calcium phosphate-rich layer forming on the Si-rich layer due to the migration of Ca^2+^ and (PO_4_)^3−^ ions; and (v) a polycrystalline apatite layer forming on the surface of the bioglass. Collagen fibers can attach to the surface of the bioactive glass. The transparent silica-rich layer induces precipitation of the hydroxyapatite-like (HCA) layer. Interactions between bioglass and collagen fibers occur and become stronger when HCA precipitation increases [187].

Guidoin et al. [188] reported that a thick collagenous tissue partly penetrated the outer layers of the braided structure of a UHMWPE prosthesis. This collagen penetration caused the expansion and separation of the multifilament yarns into individual fibers. However, while the knit fabric was encapsulated by thin collagenous tissue, there was no significant infiltration into the structure. Thus, a hollow braided structure was designed with a core of parallel poly (vinyl alcohol) (PVA) cord wrapped by the braided diamond structure of UHMWPE threads for better mechanical performance [189]. Bach et al. [35] have invented a hydrogel fiber for ACL reconstruction, made from PVA hydrogel. Tensile strength was enhanced by incorporating UHMWPE fibers around the PVA cord.

Zhang et al. [190] reported that UHMWPE filament could be modified with polycaprolactone for ligament and tendon regeneration [191]. Absorbable polycaprolactone PCL has attracted mainstream attention in recent years for the development of tendon/ligament repair materials due to its excellent performance attributes of low degradation, high stability, non-toxicity, and bioresorbability [192]. Fibrous PCL has also been reported to be able to help cell growth.

Schmidt et al. [193] reported that growth factors play an essential role in the stimulation of fibroblast division and ligament healing. Growth factors such as platelet-derived growth factor AA, platelet-derived growth factor-BB, basic fibroblast growth factor, insulin-like growth factor 1, and interleukin 1- alpha can enhance the proliferation of fibroblastic cells. Growth factors can elicit specific biological responses such as proliferation, chemotaxis, matrix synthesis, and secretion of other growth factors during wound healing. Molloy et al. [194] investigated some of the recent research into the functions of five growth factors whose actions were better defined during tendon healing. Those five growth factors are: Insulin-like growth factor I (IGF-I), Transforming growth factor β (TGF -β), Vascular endothelial growth factor (VEGF), Platelet-derived growth factor (PDGF), and Basic fibroblast growth factor (BFGF). Table 13 summarizes the role of the growth factors in tendon or ligament healing process.

**Figure 13:**
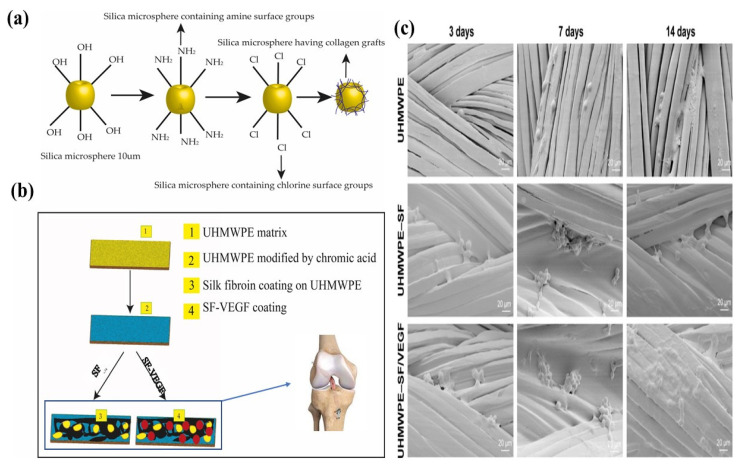
(**a**) Bioactivity of bioglass; (**b**) schematic preparation of SF/VEGF coating [37]; (**c**) morphology of different groups of UHMWPE [37].

Based on previous research studies, the biofunctionalization of UHMWPE was conducted by loading of VEGF (vascular endothelial growth factor) into UHMWPE followed by SF (Silk fibroin) coating for ACL reconstruction [37]. Firstly, UHMWPE fibers were treated with ethanol and chromic acid to remove impurities. Chromic acid introduced additional functional groups to the surface of the fibers and etched the amorphous regions of threads. The chromic acid-treated UHMWPE was then immersed in either SF or VEGF/SF solution at 4 °C for 12 h. SF loading growth factor VEGF was used to achieve the sustained release and to improve the neovascularization. Figure 13b presents the whole procedure of the SF/VEGF coating and reconstruction model. Cell morphology of bone marrow mesenchymal stem cells (BMSCs) is shown in Figure 13c. Filopodia of BMSCs attached to the surface of bare UHMWPE were not visible until after 14 days of cultivation, but it was noticed on the surface of UHMWPE–SF and the UHMWPE–SF/VEGF group after 7 days of cultivation [202].

## 6. Fixation of UHMWPE Graft Animal Models for Ligament Reconstruction

Several animal models of ACL reconstruction have been reported in different articles [203,204]. According to these methods, an inhalation mask was used to administer two percent isoflurane in O_2_ gas (1.5 L/min) to the animals. Procedures were carried out on a heating blanket in a sterile atmosphere. The animal was put in the supine position on the surgical table. The selected knee section was sanitized before skin cuts were made. The lateral parapatellar arthrotomy was utilized to uncover the knee joint of the animal. A notchplasty was performed to remove remnants of ligaments [205]. The bone tunnels were made using a 3.0 mm drill in the anatomic sites of the natural ACL in the femur and tibia. The UHMWPE grafts were threaded through the tunnels and knotted out of the femoral and tibial bone tunnels on both ends. The wound was irrigated with sterile saline solution after the graft was permanently attached. Lastly, sutures were used to seal the capsular layers and skins [206].

## 7. Conclusions

While many polymers, metals, ceramics, and composite materials are in use as biomaterials, UHMWPE is one of the most important of the bioinert polymers used in the manufacture of medical implants. Problems associated with the use of UHMWPE as implants include wear debris and oxidative degradation due to the generation of free radicals when exposed to irradiation with gamma rays for grafting or sterilization.

To resolve the issue of wear and to enhance the life of UHMWPE as an implant, in recent years this field has witnessed numerous innovative methodologies, such as biofunctionalization or high temperature melting of UHMWPE to enhance its toughness and strength. Sometimes one surface modification strategy is taken to solve a particular wear problem but may lead to a new problem and further strategy is required to eliminate that new problem. For example, the bioreaction of soft tissues is triggered by UHMWPE wear particles that can ultimately lead to aseptic loosening of hip implants. Therefore, high dose irradiation is used to highly cross-link UHMWPE which decreases the wear rate but initiates free radical formation that causes oxidative degradation in UHMWPE. To reduce or eliminate the free radical formation, annealing or post-irradiation techniques are used. Despite this, there is a chance of increased incidences of rim fracture under impingement and adverse loading conditions due to the lowered fatigue strength of this material. Thus, an alternative method of vitamin E stabilization of UHMWPE is carried out to provide oxidation resistance without sacrificing fatigue strength. However, vitamin E has a capacity to act as a free radical scavenger during irradiation which can lower the cross-linking efficiency of UHMWPE and limits the vitamin E concentration in the blend to less than 0.3 wt%.

Surface modification can improve functional properties such as mechanical properties, resistance to wear, biocompatibility, cytocompatibility, wettability, and biomaterial surface properties. Chromic acid and hydrogen peroxide can be used to reduce the smoothness of the surface, and polydopamine can be used to add functional amine groups on the surface along with protein immobilization. Recently, much attention has been focused on plasma treatment for surface modification of UHMWPE. DBD plasma was introduced to modify the surface properties of materials and then later PACVD, ECR, CAP, and PIII introduced a new era in surface modification compared to other surface functionalization methods. Plasma treatments can improve the hydrophilicity of materials, reduce the smoothness of the surface, and increase protein attachments through cross-linking and covalent bonding. It has been stated that different cellular functions such as adhesion, proliferation, and differentiation are influenced by surface energy, surface functionalization, and surface morphology. Among different plasma methods, plasma immersion ion implantation (PIII) is receiving attention due to the biofunctionalization of materials with complex shapes. In addition, it allows the use of protein immobilization. The surface functionalization of UHMWPE is quite straightforward, and surface treatments can be used to change only the surface properties without affecting the bulk properties of the material.

UV irradiation is the most common method used for cross-linking of free radicals with the substrate. UV irradiation and grafting can modify the wear and mechanical properties of material. Adverse effects of oxidation can be improved by blending vitamin E with the substrate during the treatment.

UHMWPE is a unique material due to its high capacity for vascularization throughout the whole structure, which is considered a primary requirement of grafts and other biomedical prostheses. Ligament/tendon reconstruction is considered a promising research application area for UHMWPE. Owing to the extreme hydrophobicity of UHMWPE and its surface chemistry, which is very different from that of natural ligaments and tendons, modern ACL reconstructions do not enjoy the low friction and wear of the original ligaments. Chitosan-hyaluronic acid composite has been used for modification of UHMWPE grafts. Bioglass and PCL coatings on UHMWPE show excellent results in fibroblastic cell adhesion assays and ligament regeneration. Several proteins and growth factors on the surface of UHMWPE showed significantly improved outcomes on ligament/tendon regeneration. The results described in the literature were related to the surface improvement of UHMWPE with protein adsorption making the surface bioactive for cell adhesion by displaying the signaling motifs of biological molecules. However, successful biofunctionalization depends on selection of the proper type and concentration of protein molecules to minimize inflammation, friction, and wear related issues of UHMWPE implants. Surface functionalization of biomaterials for ligaments/tendons is in need of being further developed, with the potential for improved outcomes for patients.

## 8. Challenges and Future Perspectives

Many of these procedures have been proven to be successful in the laboratory by competent chemists, but that is not the case in manufacturing. The essential chemicals are potentially dangerous, costly, or currently unavailable in the large quantities required for manufacturing. Plasma treatment for polymer surface alteration has acquired an amazing consideration, attributable to its potential benefits in improvement of surface properties without influencing mass properties. Yet, non-uniformity, instability, inhomogeneity, and transfer into hot plasma over long treatment periods remain challenges to some plasma treatment. The integration of two or more modification methods revealed fascinating multi-functional characteristics; however, due to complex methodology and high cost, this strategy does not appear to be scalable.

Significant progress has been made in the field of functionalization of UHMWPE implants for ligament/tendon regeneration. Biological functionalization of orthopedic surfaces is a well-studied field with a lot of opportunities for advancement. The adaptation of current biomolecule immobilization techniques is the challenge for the next generation of research in this subject. Among different plasma treatments, plasma immersion ion implantation showed promising results due to covalently binding biomolecules to the surface of UHMWPE. A better understanding of the surface chemistry of UHMWPE with proteins is needed for more innovative biomolecule selection and design, potentially resulting in more effective multifunctional interfaces. If this is achieved, it will be a major step forwards in the development of this fiber for a variety of high-performance applications.

## Figures and Tables

**Table 1:** The influence on surface properties of UHMWPE after surface treatment.

Treatment	Parameters	Additional Peaks Found after Treatment	Elemental Analysis after Oxidation on the Surface (%)				Surface and Tribological Properties	Mechanical Properties	References
			C	O	N	Others			
Chemical	Chromic acid, hydrogen per oxide, potassium permanganate		89.6, 73.6, 91.6	9.6, 16.5, 6.6	-		Improvement in surface adhesion properties	Improvement in interfacial tensile strength	[40]
Chemical	Polydopamine, Ethylene glycol diglycidyl ether (EGDGE)						Water contact angle (WCA) decreased from 109° to 97°	67.5% improvement in mechanical strength	[41]
Chemical	Polydopamine (PDA), hexamethylene diamine (HMDA)	C-OH, -NH, C=C stretching	73.14, 74.39, 74.39	21.3, 18.65	5.13, 6.96			Shear strength 0.920 MPa	[42]
Chemical	Hyaluronic acid						WCA decreased from 80° to 50°, increased crystallinity	High wear resistance	[43]
Chemical	PEG-like coating	-OH stretch, C-O-C, C-OH	68.8	31.2	-		WCA decreased from 90.3° to 44.8°		[44]
Chemical	VTMS and SiO_2_	Si-C, Si-O	40.39	35.38		24.23	WCA decreased from 123° to 43°, increased antifouling properties		[45]
Cold plasma	He and O_2_ gas	C-C stretching, C-N, Hydrogenated amorphous C					Increased surface hydrophilicity and cell adhesion	High wear resistance	[46]
Cold plasma	H_2_ and O_2_ gas	CH vibrations, C-O stretching, C=O					WCA decreased from 102° to 43°, Improved cell adhesion property, Roughness increased from 588 nm to 687 nm		[46]
PACVD plasma	Air and Ar gas							Tear resistance, 40% increase in tensile strength	[22]
ECR plasma	H_2_ and N_2_ gas	C-O/C-OH, C-C, C=C, C=O	60	37	8		Increased surface roughness	Increased surface hardness and elastic modulus	[33]
ECR plasma	(N_2_ + H_2_) and O_2_ gas	C-N, C-O Stretching, N-H bending, C=O Stretching					WCA decreased from 96° to 22°, improvement in surface cell adhesion		[21]
ECR plasma	Peroxides, acrylic acid, itaconic acid, collagen	C-O, -COOH, -NH, C-N							[34]
DBD + chitosan treatment	(Ar + O_2_) gas and chitosan	C=O, -COO, C-O/C-N	93.15, 82.1, 74.0	5.8, 15.2, 2.7	1.1, 2.7, 3.0		Surface adhesion increased by 72.2%, WCA decreased from 101° to 82°	Decreased tensile strength by 5.6%	[47]
DBD plasma	(He + Ar + air + N_2_ + H_2_) gas	C-C, C-C=O, O=C-O	88.3	6.3	6.9		WCA decreased from 95.2° to 70°		[48]
DBD plasma	Ar gas	C-H, -OH, C-C, C=O					WCA decreased from 91° to 67.4°		
DBD plasm	Ar gas, Multi walled carbon nanotube	C-H stretching, C-C, O-H, C=O					Wear volume was reduced by 73.3%, surface roughness was reduced by 17%, hardness of the composite increased by 45%		[30]
Gamma	Thermal treatment + gamma dose							Toughness increased by 67%,	[49]
Gamma							Increased cross-linking, wear rate 37%		[50]
Gamma	UV + PEG grafting	C=C, C-Si, C=O					Crystallinity increased, resistance to protein adsorption increased		[51]
Gamma	Irradiation + Vitamin E						Reduced crystallinity, wear resistance		[52]
PIII treatment	Ar gas	C=C, -OH, COOH and COO-					WCA decreased from 80° to 28°		[53]
PIII treatment	N_2_ + gas						A small decrease in WCA, surface roughness increased from 39 nm to 71 nm		[54]
PIII treatment	N_2_ + gas + HRP protein		71.1	15.5	13.4		Roughness increased from 528° to 1130°, surface area increased from 101.9 nm to 15.6 nm		[55]
PIII treatment	N_2_ + HRP + PVC	C=O, C-O, -OH					WCA decreased from 90° to 58°		[56]

**Table 2:** Physical properties of HDPE and UHMWPE [3].

Property	HDPE	UHMWPE
Molecular weight (×10^6^ g/mol)	0.05–0.25	3.5–7.5
Tensile ultimate strength (MPa)	22–31	39–48
Tensile ultimate elongation (%)	10–1200	350–525

**Table 3:** Elemental analysis of UHMWPE after oxidation on the surface [40].

Scheme	C (%)	O (%)
Untreated	74.2	21.9
Chromic acid	89.6	9.6
Potassium permanganate	73.6	16.5
Hydrogen peroxide	91.6	6.6

**Table 4:** Adhesion properties of DLN films on UHMWPE substrate [96].

Ar Gas Flow (mL/min)	LP (w)	Adhesion Strength (MPa)	Load Applied (N)
25	180	12	65.8
50	225	33	187.3
75	290	37	209.2
100	260	20	112.6
175	255	12	70.6

**Table 5:** Mechanical strength comparison of untreated and treated UHMWPE [41].

Samples	Amount of Fibre (vol%)	Tensile Strength MPa
Raw UHMWPE	34.6	526.7
DBD-UHMWPE	34.2	543.4
BHHBP-DGEBA-UHMWPE	34.5	537.3

**Table 6:** Coefficient of wear and friction [117].

Sample	Coefficient of Wear (mm^3^/Nm)	Coefficient of Friction
UN	19.6 × 10^−9^	0.060–0.063
PE1	14.1 × 10^−9^	0.066–0.068
PE2	8.45 × 10^−9^	0.065–0.066
PE3	6.51 × 10^−9^	0.060–0.070

**Table 7:** Total surface energy, polar, and dispersive components in PIII treated UHMWPE [118].

Sample	Polar Components (dyne/cm)	Dispersive Components (dyne/cm)	Total Surface Energy (dyne/cm)
Untreated	18.7	39.5	58.2
PIII treated	50.7	22.1	72.8

**Table 8:** Disadvantages of traditional parameters used for surface functionalization methods [118].

Parameter	Effects
High temperature	Deteriorates the material [119]
High-intensity energy	Oxidizes the material, increases crystallinity, destroys the lifetime [120]
UV radiation	Oxidative degradation of the material can damage the material [17,60]
Chemical reagent	Changes surface chemistry, the formation of toxic residues and by-products including carcinogens [36]

**Table 9:** Advantages and disadvantages of different plasma methods [61,121,122,123].

Methods	Advantages	Disadvantages
DBD plasma	Atmospheric pressure Less time required	Sample could be contaminated
PACVD plasma	High deposition rate Low temperature Unique chemical properties of deposited film High solvent and corrosion resistance	Time consuming Unstable against ageing Very thin layer of deposition
ECR plasma	Low-pressure range High degree of ionization Provide sterile environment	Non-uniform etch surface Sample could be contaminated
Cold plasma	Can provide sterile environment Low temperature Suitable for porous structures Uniform surface treatment	Covers limited surface area Limited temperature range
PIII	Large treatment area High efficiency Can treat 3D objects Batch processing capability Small instrument footprint Provide uniformity in treated surface	Difficult to achieve accurate in situ dose monitoring

**Table 10:** Surface property influence on HRP immobilization after PIII treatment on UHMWPE.

Sample	Roughness A°	Surface Area (µm^2^)	C (%)	N (%)	O (%)	Optical Density @450nm from HRP Activity
Untreated	528	101.9	94.9	Nil	5.1	0.45
PIII treated	1130	105.6	71.1	13.4	15.5	0.52

**Table 11:** Protein attachment on UHMWPE with or without additives [56].

Samples	HRP Protein Remains after Wash (%)
Untreated	19
PIII treated	84
PIII treated + Plasticizer	62–72

**Table 12:** Mechanical properties of materials for synthetic grafts compared to normal ACL [168].

Grafts	Ultimate Tensile Load (N)	Stiffness (N/mm)
Human ACL	1730	242
Human hamstring graft	3790	776
The human patellar tendon graft	3790–4140	685
Carbon fibers	660	230
Gore-Tex prosthesis	5300	322
Dacron	3631	420
Twisted silk matrix	2337	354
Parallel silk matrix	1740	2214
KLAD	280	1500
Trevira	68.3	1866
Leeds-Keio	270	2000
UHMWPE fabric	52570	115

**Table 13:** A summary of the role of the seven growth factors during tendon and ligament healing.

Growth Factor	The Active Site of Growth Factors	Role	Reference
PDGF AA	Proliferation, remodeling	Controls DNA and protein synthesis at the injured site, controls the expression of other growth factors.	[193]
PDGF BB	Proliferation, remodeling	Controls DNA and protein synthesis at the injured site, controls the expression of other growth factors.	[195]
IGF-1	Inflammation, proliferation	Supports the proliferation and migration of cells, triggers matrix production.	[196]
TGFβ	Inflammation	Controls cell migration, proteinase expression, fibronectin-binding interaction, and stimulation of collagen production.	[194]
VEGF	Proliferation, remodeling	Supports angiogenesis.	[194]
bFGF	Proliferation, remodeling	Supports cellular migration, angiogenesis.	[193]
IL1A	Proliferation induces pro-collagen type I and III synthesis	Supports proliferation, and induction.	[197]
BMP	Remodeling of impaired tissues	Supports angiogenesis.	[198]
GDF	Proliferation	Supports in ligament/tendon formation.	[198]
Elastin	Proliferation	Controls DNA and protein synthesis at the injured site.	[199]
Heparin	Proliferation	Supports the release of growth factors.	[200]
PRP (Platelet-rich plasma)		Increased cellular metabolic activity, reduced apoptotic rate, and stimulation of collagen production in the cells.	[201]

## Data Availability

Not applicable.
